# Successful Reconstruction of Extensive Lower Lip Squamous Cell Carcinoma: A Case Report

**DOI:** 10.7759/cureus.77468

**Published:** 2025-01-15

**Authors:** Mariana Cebotari, Isabel Vazquez, Rita Silva, Martina Leão, Teresa Burnay

**Affiliations:** 1 Maxillofacial Surgery, Centro Hospitalar Universitário de São João, Porto, PRT

**Keywords:** lower lip, malignant tumor, primary wound closure, reconstruction, squamous cell carcinoma (scc)

## Abstract

This article presents the case of an 81-year-old male referred to the maxillofacial department for extensive lower lip squamous cell carcinoma (SCC). While SCC of the lip is among the most easily detectable and treatable malignancies, surgical treatment can sometimes lead to significant defects, impacting both lip function and facial aesthetics. The patient underwent a comprehensive W-type excision and primary closure, complemented by a right suprahyoid neck dissection. This case report emphasizes the successful reconstruction of a large defect using primary closure, achieving both functional and aesthetic preservation. During follow-up, the patient demonstrated optimal healing, with intact function, satisfactory cosmetic outcomes, and no clinical or imaging evidence of recurrence or distant metastasis.

## Introduction

Lip cancer predominantly affects fair-skinned individuals with a history of prolonged UV radiation exposure or acute childhood sun damage, such as sunburn [1,2]. Additional risk factors include tobacco use, alcohol abuse, certain viruses (especially in immunocompromised individuals), and demographic characteristics like age and sex, with males over 45 years being most commonly affected [1-4]. While lip carcinoma has a low overall incidence (1-2%) [4], it accounts for 23.6-30% of all oral malignancies [1]. These carcinomas often develop from precancerous lesions, such as actinic cheilitis - a chronic inflammatory condition with potential for malignant transformation [1,4]. Early diagnosis and treatment of these lesions are critical to preventing their progression into malignant tumors [1,4].

Squamous cell carcinoma (SCC) is the most common malignant tumor of the lip, followed by basal cell carcinomas and, less commonly, adenocarcinomas arising from minor salivary glands, melanomas, sarcomas, and lymphomas [4]. SCC frequently occurs on the lower lip and has the potential to metastasize to the neck [4]. Factors such as tumor size (greater than 2 cm), patient age (under 40 years), histological grade, and perineural invasion are associated with a higher risk of local recurrence and distant metastases [1,4,5].

A biopsy is crucial for confirming the diagnosis of carcinoma. In early stages, the risk of cervical lymph node metastasis is approximately 8%, but this risk increases significantly in advanced cases [4]. Imaging studies, including CT or MRI, are routinely used for diagnosis and treatment planning.

Treatment options depend on tumor characteristics and may include surgery or radiotherapy. Surgical excision requires meticulous planning to balance oncological clearance with functional and aesthetic preservation [1,4]. Comprehensive surgical management often includes full-thickness tumor excision, lymph node dissection, and reconstruction. While elective neck dissection is recommended for evident neck involvement (clinical or radiological), its role in treating N0 neck remains debated [1,4].

Reconstruction methods vary depending on the defect size. Lesions involving up to one-third of the lower lip are typically treated with V-type or W-type excisions, whereas larger defects (up to two-thirds of the lip) may necessitate local or free flaps for reconstruction [1,4].

## Case presentation

**Figure 1:**
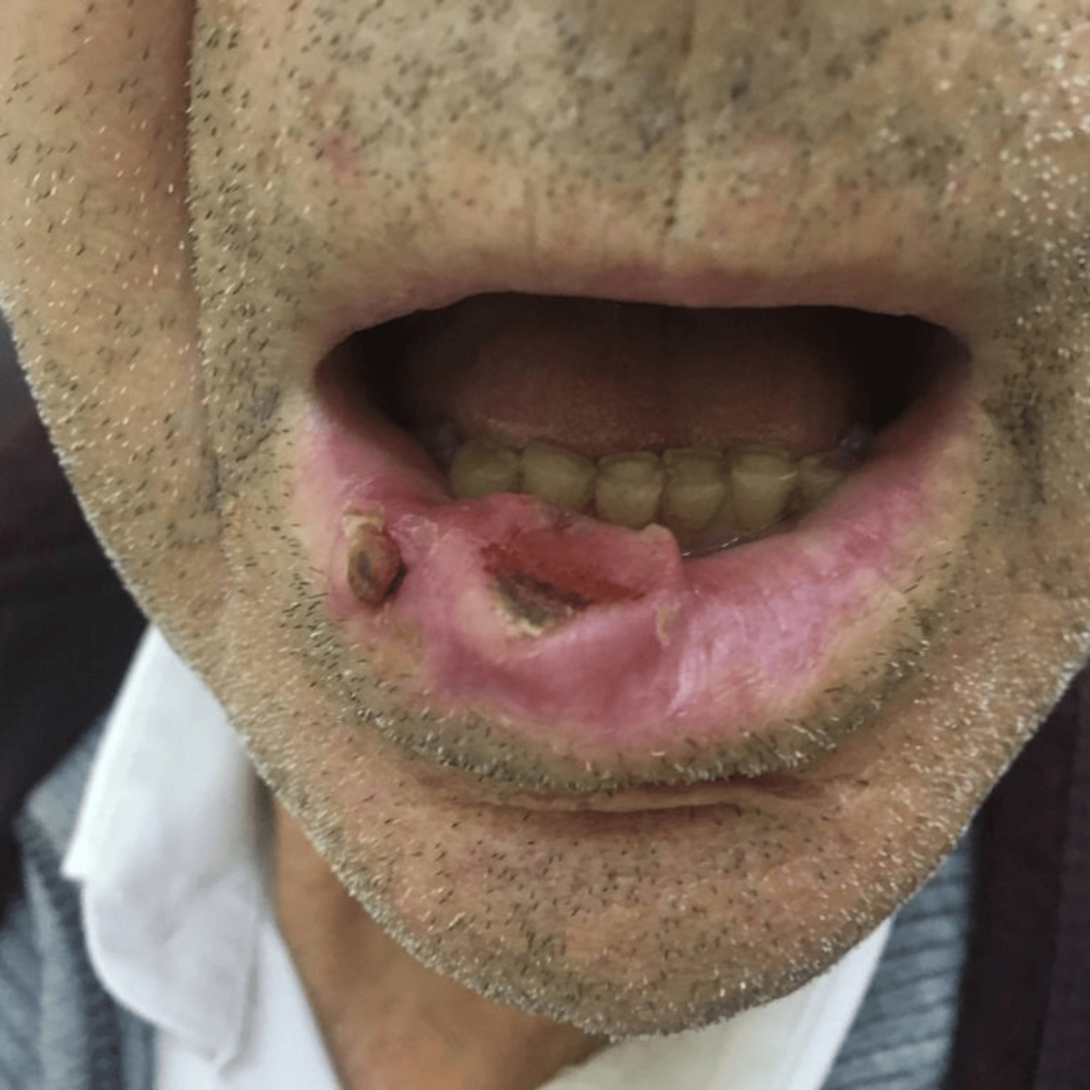
Ulcerated lesion affecting the right and central regions of the lower lip

An 81-year-old male was referred to the maxillofacial department due to a lower lip lesion that had been gradually growing for over a year. At the time of presentation, the patient did not report any symptoms, such as pain, difficulty speaking, or trouble eating. His medical history included hypertension, diabetes, and epilepsy. He denied any history of tobacco or alcohol abuse. The patient had worked as a civil construction worker until his retirement. On examination, he presented with an ulcerated lesion involving the right and central areas of the lower lip, measuring approximately 2.5 cm (Figure 1), with no palpable cervical nodes or masses.

**Figure 2:**
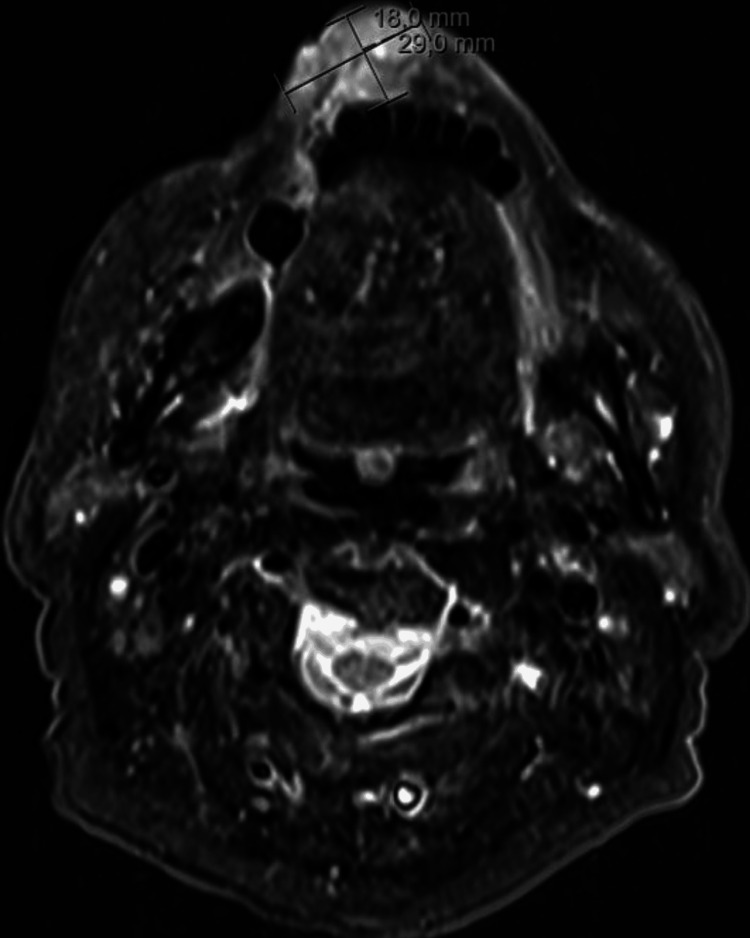
MRI scan revealing a 29 × 18 mm mass in the lower lip

**Figure 3:**
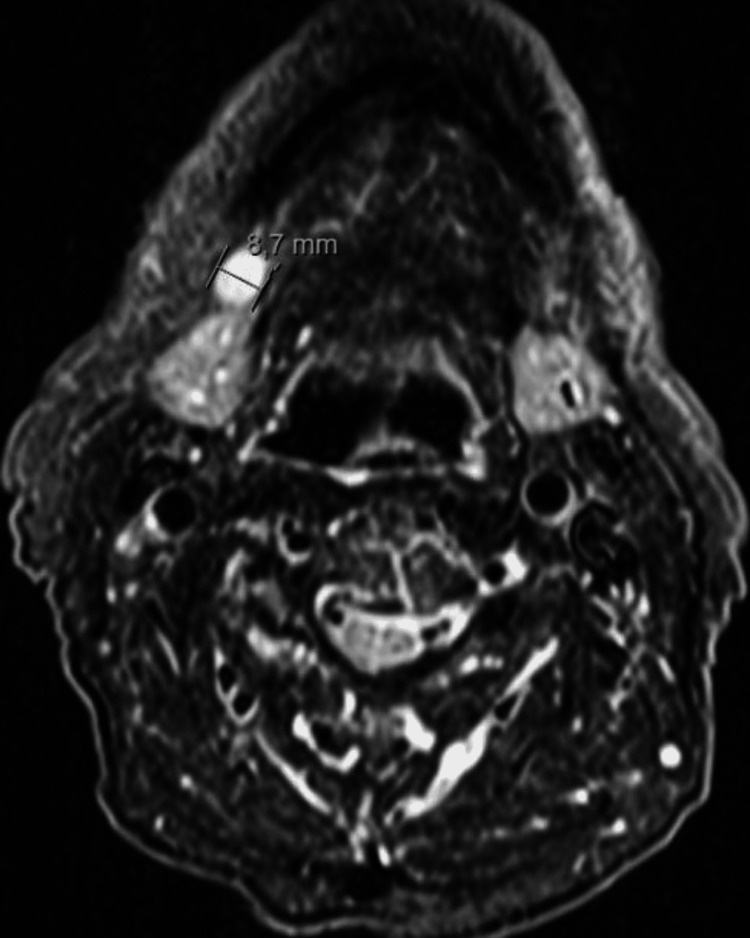
MRI scan highlighting the largest right level I lymph node

An MRI was performed, revealing a 29 × 18 mm mass in the lower lip (Figure 2) and enlarged right level I lymph nodes, with the largest measuring 9 mm in diameter (Figure 3).

**Figure 4:**
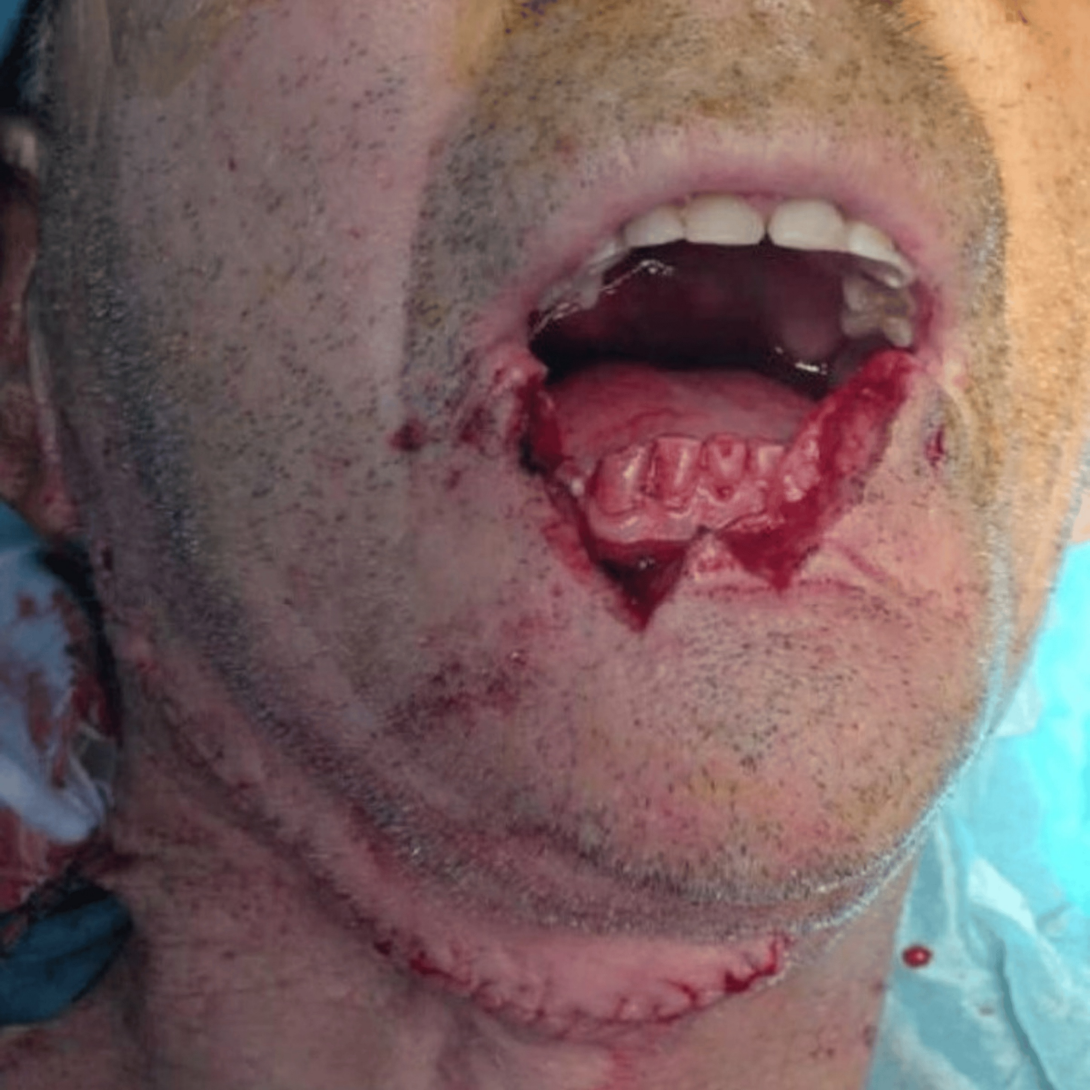
Defect resulting from tumor excision

The patient underwent an extensive W-type excision (Figure 4) with primary closure, along with a right suprahyoid neck dissection. The margins of the specimen sent for intraoperative histopathological evaluation were free of tumor.

**Figure 5:**
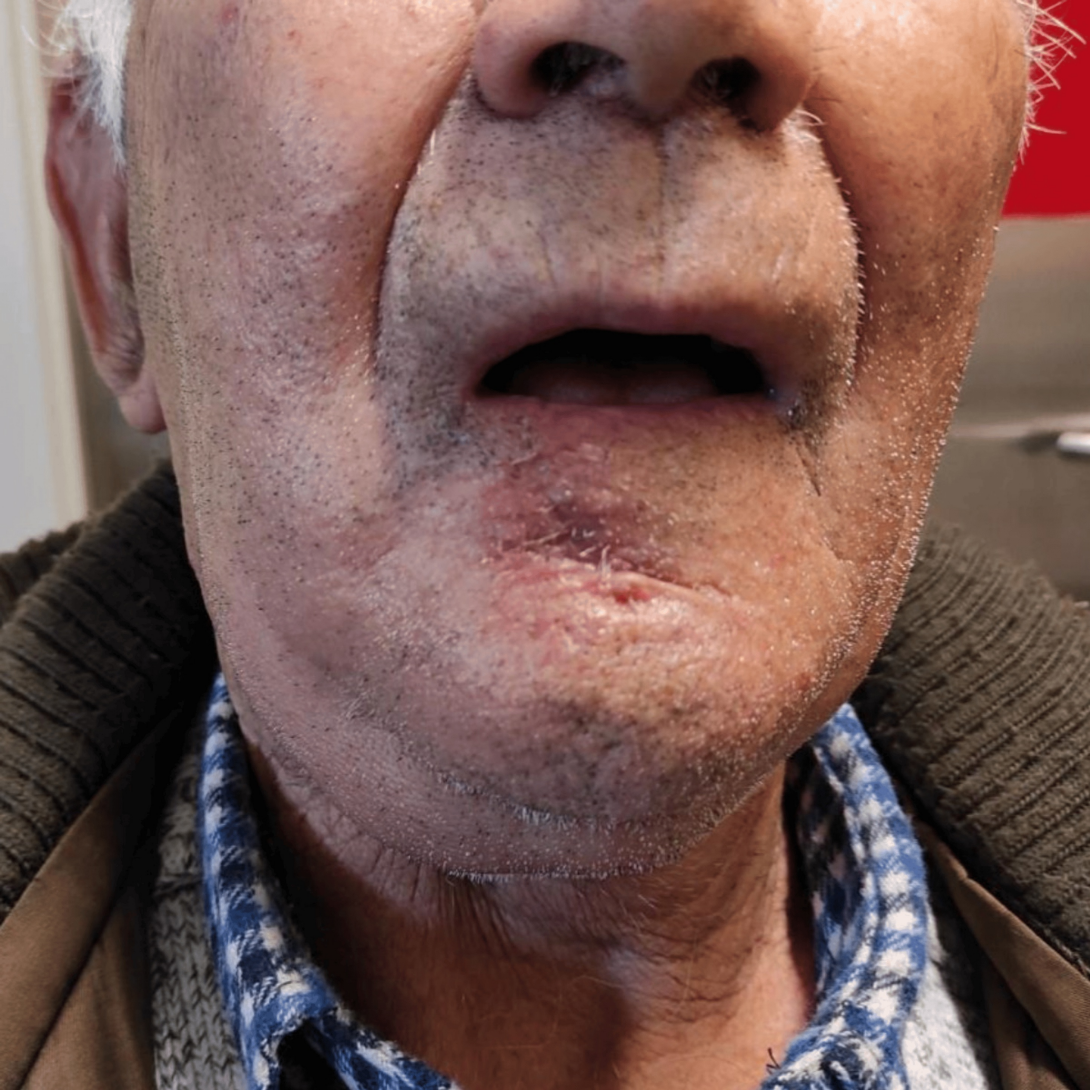
One month post-surgery

**Figure 6:**
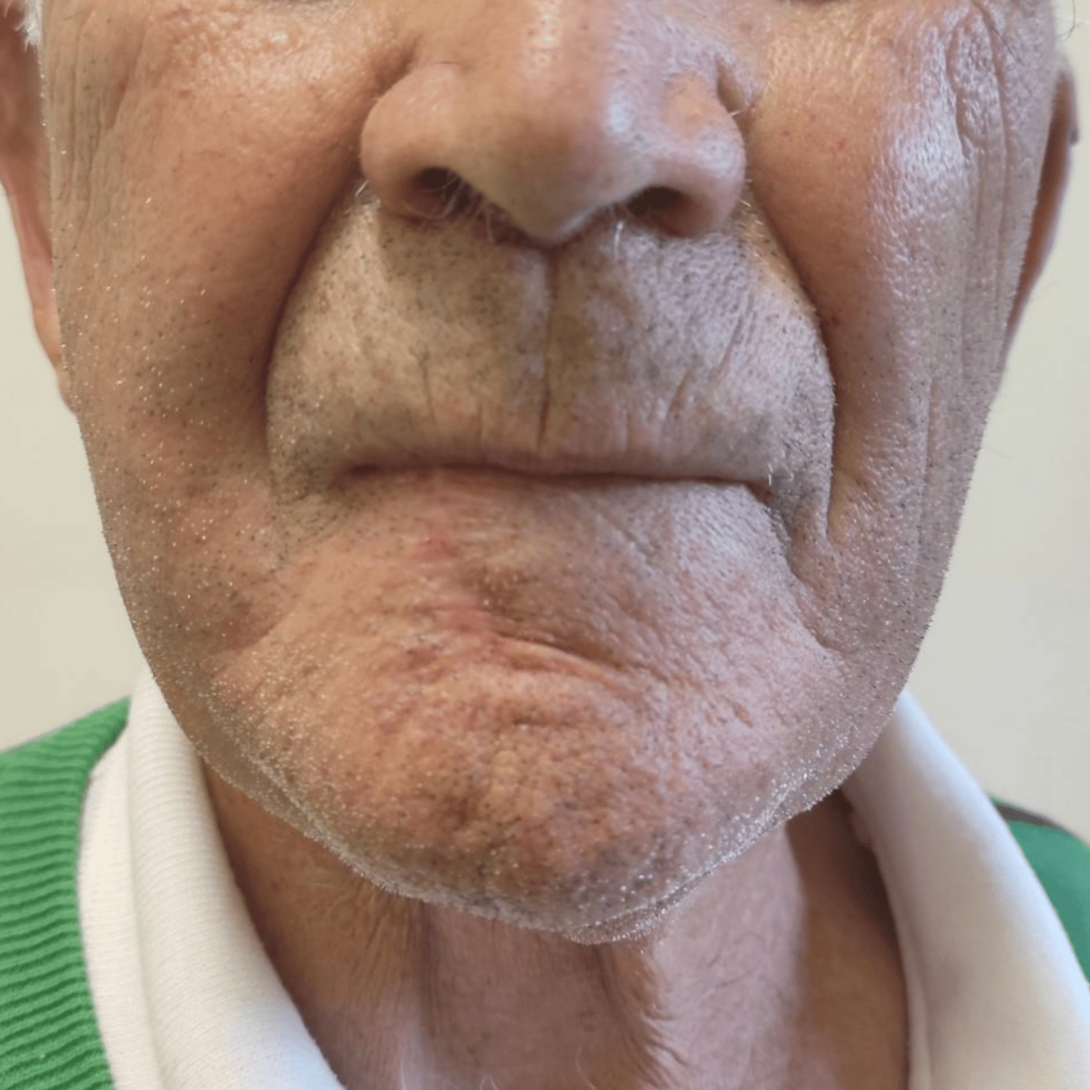
Six months post-surgery

The final histopathological report confirmed a moderately differentiated SCC, classified as pT2N0. The case was discussed in a multidisciplinary team meeting, and no further treatment was deemed necessary. A close follow-up was decided. During follow-up, the patient demonstrated optimal healing, with preserved function and satisfactory aesthetic results (Figure 5, Figure 6).

The patient experienced some postoperative discomfort, which resolved quickly with oral medication. He maintained preserved oral competence and did not require physical rehabilitation. During follow-up, there were no clinical or imaging signs of recurrence or distant metastasis.

## Discussion

SCC is the most common malignant tumor of the lip, typically affecting men and predominantly involving the lower lip [1,4]. While several factors contribute to the risk of SCC, cumulative sun exposure - particularly during childhood and adolescence - is the most significant. Some risk factors are associated with an increased likelihood of local recurrence and distant metastases [1-5]. Although SCC is usually diagnosed and treated in its early stages, delayed treatment can lead to substantial tumor growth and damage to surrounding healthy tissue [1].

The TNM system is commonly used for disease classification and treatment planning, as both T and N stages are significant prognostic indicators in various studies [4]. Surgical full-thickness tumor resection with negative margins is the cornerstone of treatment for lip cancer. Tumor size and neck involvement are important factors in determining the most appropriate treatment approach [1,4]. A key area of controversy is the management of the neck. While many authors advocate for elective neck dissection in cases of evident neck involvement (clinical or radiological), the treatment of N0 neck remains debated [1]. The five-year survival rate for lip cancer is approximately 90%, but this drops to around 50% in cases involving lymph node metastasis [1,4]. Recurrence rates vary based on factors such as tumor size, location, previous treatment, and histology, highlighting the importance of thorough follow-up [1,4].

The primary goal of lower lip reconstruction is to restore oral competence and aesthetic integrity. Lesions involving up to one-third of the lower lip are generally managed with V-type or W-type excisions and primary closure [1,4]. Larger defects pose a greater challenge for the surgeon, requiring consideration of the patient’s general health, comorbidities, and desired functional and cosmetic outcomes. Patient involvement in the decision-making process is essential. For defects greater than one-third of the lower lip with adequate remaining tissue, reconstruction can be performed using local flaps and techniques such as those described by Karapandzic and Abbe [6,7]. Larger defects, including soft tissue or bony loss, may require local or free flaps for reconstruction.

Our patient presented with an extensive lower lip lesion. However, due to advanced age and comorbidities, we opted for a W-shaped excision followed by primary closure, achieving excellent functional and aesthetic results. This approach can be applied in selected cases, as demonstrated in this report.

Follow-up schedules may vary according to national guidelines. Our patient underwent follow-up consultations at one, three, and six months, and again at one year post-surgery. Thereafter, follow-up occurred every six months during the second year, with annual consultations thereafter. During these visits, physical examinations showed no clinical signs of recurrence or metastasis. MRI and cervical ultrasonography also revealed no imaging evidence of recurrence or metastasis.

## Conclusions

Lip cancer remains one of the most treatable malignancies in the head and neck region, with surgery playing a central role in its management. The extent of the surgical defect determines the reconstruction approach, but it is crucial to also consider the patient’s overall health and expectations.

This case underscores the importance of selecting the most appropriate treatment plan for each patient and serves as a valuable reference for similar cases in the future. Despite the extensive lesion involving half of the lower lip, we opted for primary closure, achieving excellent aesthetic results and, most importantly, preserving oral competence. This approach should be considered, as it offers advantages over local or free flaps, including reduced scar tissue, better cosmetic outcomes, and no risk of donor site morbidity.
